# Changes in the expression of MMP2, MMP9, and ColIV in stromal cells in oral squamous tongue cell carcinoma: relationships and prognostic implications

**DOI:** 10.1186/1756-9966-31-90

**Published:** 2012-10-29

**Authors:** Hai-Xia Fan, Hai-Xia Li, Dong Chen, Zhong-Xiuzi Gao, Jin-Hua Zheng

**Affiliations:** 1 Department of Anatomy, Basic Medical Science College, Harbin Medical University, Harbin, China; 2 Harbin Medical University Stomatological Hospital, Harbin Medical University, Harbin, Heilongjiang, China; 3 Department of Physics and Laboratory of Sono- and Photo-theragnostic Technologies, Harbin Institute of Technology, Harbin, China; 4 Department of Anatomy, Basic Medical Science College of Harbin Medical University, 194 Xuefu-Road, Harbin 150081, China

**Keywords:** Oral tongue squamous carcinoma, MMPs, ColIV, Immunohistochemistry, Prognosis

## Abstract

**Background:**

Type IV collagen (ColIV) is the most important scaffold for the basement membrane (BM) proteins, and plays an important role in regulating and limiting tumour invasion and metastasis.

**Methods:**

Here, we observed the changes in morphology and distribution of type IV collagen (ColIV) in the basement membrane (BM) surrounding nests of carcinoma in 48 patients with oral tongue squamous cell (OTSCC). We examined the correlation between the expressions of ColIV, MMP-2 and MMP-9 and the prognosis of OTSCC patients. The intensity and patterns of expression were assessed immunohistochemically using anti-human mouse monoclonal MMP-2, MMP-9 and Col IV antibodies. Statistical analyses were performed to determine the prognostic correlations of ColIV, MMP-2, and MMP-9 levels.

**Results:**

MMP-2 and MMP-9 expressions in OTSCC were higher than those in normal oral mucosa and dysplastic oral mucosa group(MMP-2 iOD: 66.40 ± 24.20, 134.69 ± 37.08, and 357.79 ± 116.78; MMP-9 iOD: 88.05 ± 23.85, 307.13 ± 93.22, and 791.31 ± 260.52; in normal, dysplastic oral mucosa, and tumour tissues, respectively, P < 0.01); however, ColIV immunoreactivity was lower (ColIV iOD: 406.87 ± 62.95, 247.83 ± 42.30, and 151.92 ± 38.17 in normal, dysplastic oral mucosa, and tumour tissues, respectively, P < 0.01). High tumour and stromal MMP-2 and MMP-9 expression was significantly associated with positive lymph node status. Col IV expression was associated with positive lymph node status (P < 0.05), and have negatively correlated with the expression of MMP-2 and MMP-9. Overall survival was significantly shorter in patients with high tumour and stromal MMP-2 and MMP-9 expression, and tended to be shorter in patients with low ColIV expression.

**Conclusions:**

Degradation of ColIV was closely related to increased MMP-2 and MMP-9 expression; MMP-9 have more important function than MMP-2 during the cancer development. Monitoring changes in the expression of ColIV, MMP-2, and MMP-9 may be a useful technique for assessing prognoses in OTSCC patients.

## Background

Oral tongue squamous cell carcinoma (OTSCC) is the most common malignancy diagnosed in the oral and maxillofacial regions [1], which is characterized by a high degree of local invasiveness and a high rate of metastasis to cervical lymph nodes [2]. Notably, infiltration is a prerequisite and key step of cancer metastasis; and is an important factor in the prognosis of patients with oral cancer. Therefore, predictions of tumour infiltration and metastasis, and prognosis based on clinical parameters are of great clinical importance.

A key step in OTSCC infiltration and metastasis is the degradation of the basement membrane (BM) between the epithelium and lamina propria, around cancer nests, and surrounding vascular structures [3-5]. Type IV collagen (ColIV) is the most important scaffold for the BM proteins [6], and helps maintain continuity and integrity of the BM. Tongue squamous cell carcinoma is prone to infiltration, during which ColIV in and around epithelial, vascular and tumour BM is often damaged, thus compromising its ability to limit the tumour invasion and metastasis [7-9].

High levels of proteases and breaching of BM are key stages of cancer invasion [10]. High levels of proteases facilitate degradation of BM and extracellular matrix (ECM), thus providing channels that allow tumour cells to migrate and metastasize the vascular and lymphatic systems [11]. Furthermore, the invasiveness is associated with the ability of these proteases to degrade the BM [12].

The matrix metalloproteinase (MMP)-2 and MMP-9 are gelatinases, also called type IVcollagenases [13]. They mainly degrade ColIV, the main component of BM and ECM; they also play a role in neovascularization [14]. Various matrix metalloproteinases (MMPs) are secreted during the growth, invasion, metastasis, and angiogenesis of tumours, and affect the surrounding microenvironment, causing dynamic changes [15]. Because ColIV is widely distributed in tongue tissue, its physiological and pathological significance in OTSCC has gradually attracted much attention.

Therefore, research on the MMPs that mediate invasion and metastasis of tongue cancer and the distribution and morphology of ColIV in and around epithelial and tumour BM is very necessary. In our present study, we aimed to investigate the expression of MMP-2, MMP-9 and ColIV, and the changes in the morphology of ColIV during tongue cancer development and their relationship with the stage and differentiation of OTSCC in order to determine if these results can be used to assess the prognosis in OTSCC patients.

## Materials and methods

### Patients

We collected 48 tissue samples from OTSCC patients diagnosed and treated at the Harbin Medical University Stomatological Hospital, Harbin, Heilongjiang, China, from the year 2000 to 2005. All specimens were obtained in accordance with the applicable ethical and legal standards. All patients underwent potentially curative surgery without preoperative therapy. The clinical and pathological characteristics of these patients are summarized in Table 1. Non-cancerous tissue samples (normal group and dysplastic oral mucosa group) were obtained from the tissue 2.0–2.5 cm away from the primary tumour [16], and graded its organization according with the tissue morphologically. After treatment, all the patients were followed up until death or for at least 60 months. All the patients were staged according to the 1997 UICC TNM Classification of Malignant Tumours [17].

**Table 1:** Patient characteristics

**Characteristic**	**Number**	**%**
	**(N = 48)**	
Gender		
Male	36	75.0
Female	12	25.0
Age		
<55	20	41.7
≥55	28	58.3
Differentiation		
Well-differentiation	24	50.0
Moderately	20	41.7
Poorly	4	8.3
Clinical stage		
I	10	20.8
II	2	4.2
III	21	43.7
IV	15	31.3
T-stage		
T1	22	45.8
T2	23	47.9
T3	1	2.1
T4	2	4.2
Recurrence		
No	33	68.7
Yes	15	31.3
Lymph node involvement		
No	11	22.9
Yes	37	77.1

### Immunohistochemistry

Formalin-fixed paraffin-embedded samples were sectioned at 5-μm thickness and stained with H&E for tumour confirmation. Sections adjacent to the H&E staining were used for immunohistochemical staining.

Monoclonal antibodies against MMP-2 (MAB-0244), MMP-9 (MAB-0245), and ColIV (MAB-0025) were all purchased from MaiXin Biological Technology Corporation Ltd. (Fujian, China). The concentrations of the primary antibody were 1:20 for MMP-2, 1:30 for MMP-9, and 1:100 for ColIV. The antibody was diluted with an antibody diluent.

Immunohistochemical staining was performed by using the universal two-step method [18]. Briefly, the sections were first deparaffinized with xylene and rehydrated in graded ethanol. Endogenous peroxidase activity was blocked by immersion of slides in 3% hydrogen peroxide. 1% bovine serum albumin (BSA) was applied for 15 min for blocking non-specific antigens. The mixtures were then incubated with the respective primary antibodies overnight in a humidified chamber maintained at 4°C. Subsequently, they were incubated with the corresponding secondary antibody (PV6002, Zhongshan Goldenbridge Biotechnology, Beijing, China) for 30 min at 37°C. The antibody reaction was visualized by using diaminobenzidine (DAB) chromogen (Zhongshan Goldenbridge Biotechnology). Then, all the slides were counterstained with haematoxylin. Sections incubated with immunoglobulins of the same species at the same final concentrations served as negative controls, and placental trophoblastic cells (MMP-2,-9) and bronchial epithelial cells (ColIV) were used as positive controls.

### Evaluation of immunohistochemical results

All samples were reviewed by two independent investigators who were blinded to the clinical outcomes of the patients. Image Pro Plus 6.0 (Media Cybernetics Inc.) was used to calculate the intensity of the detected molecules. Three microscopic fields in tumour tissues (original magnification 400×) were randomly selected and the integral optical density (iOD) of MMP-2, MMP-9 and ColIV was calculated by image, which was considered as the expression level of positive-staining. Higher iOD values represented higher antigen expression, and vice versa. All iOD values were divided into four quartiles as follows: 0–25%, negative expression; 25–50%, weak expression; 50–75%, moderate expression; and 75–100%, strong expression. For statistical analysis, the patients were classified into two groups: ‘low expression’ included those with negative or weak expression and ‘high expression’ included those with moderate or strong expression.

### Statistical analysis

Statistical analyses were performed with SPSS software version 18.0 (SPSS Inc., Chicago, IL). The expression of MMP-2, MMP-9 and ColIV in normal oral mucosa, dysplastic oral mucosa and OTSCC tissues were expressed as the mean ± standard deviation. The association between the clinical parameters and immunohistochemical results was analyzed with the chi-square or Fisher’s exact test (if N < 5). Survival analysis was performed using Kaplan–Meier survival curves and the log-rank test. Spearman’s rank correlation coefficient test was applied for examining the correlations among the expressions of MMP-2, MMP-9 and ColIV. P-values < 0.05 were regarded to be statistically significant.

## Results

**Figure 1:**
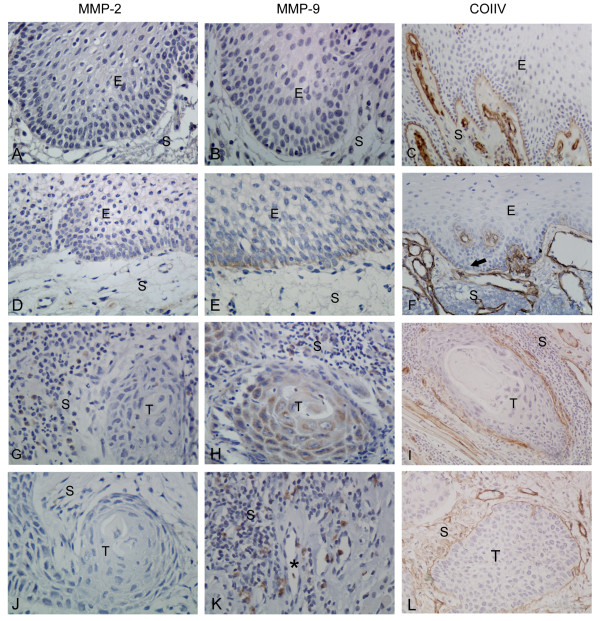
**Comparative immunolocalization of MMP-2, MMP-9 (magnification: 400×) and ColIV (magnification: 200×) in normal group, dysplastic oral mucosa group and OTSCC (T and S indicate the tumour and stroma respectively).(A, B)** The expression of MMP-2 and MMP-9 in normal tongue mucosa epithelium are negative. **(C)** Continuous expression of ColIV in the BM adjacent to basal cells. **(D)** In dysplastic oral mucosa group, the expression of MMP-2 in the basal cell layer is increased. **(E)** MMP-9 expression is mainly located in the basal cell layer of dysplastic oral mucosa. **(F)** Fragmented expression of ColIV in the BM of dysplastic oral mucosa (black arrow). **(G)** In the OTSCC tissues, MMP-2 expression are mainly located in the stromal cells surrounding the nests of carcinoma. **(J)**. In well-differentiated nests of carcinomas, the expression of MMP-2 was negative or weak positive. **(H)** The diffuse expression of MMP-9 are mainly showed in tumour and stromal cells. **(K)** MMP-9 positive cells were also accumulated around the blood vessels. **(I, L)** In the OTSCC, the expression of ColIV are showed fragmented or collapsed **(I)** and thick **(L)**.

The immunohistologic expressions of MMP-2, MMP-9 and ColIV in normal oral mucosa group, dysplastic oral mucosa group and OTSCC tissues group are shown in Figure 1.

### The expression of MMP-2, MMP-9 and ColIV in normal oral mucosa group

Positive expression of MMP-2 and MMP-9 was mainly observed in the cytoplasm of stromal cells and proliferating epithelial cells as brownish granules under 400×. Positive staining was also noted in fibroblasts, microvascular endothelial cell cytoplasm. The positive-staining cells were flaky, spotty, or scattered. The expression of MMP-2 and MMP-9 in normal tongue mucosa epithelium was negative or weak positive (MMP-2: iOD 66.40 ± 24.20, Figure 1A; MMP-9: iOD 88.05 ± 23.85, Figure 1B). ColIV in the normal tongue mucosa, adjacent to basal cells, was observed as a continuous linear structure (ColIV: iOD 406.87 ± 62.95, Figure. 1C, Additional file 1: Figure S1 A). Further, the surrounding blood vessels also tested positive for ColIV, showing a similar linear structure.

### The expression of MMP-2, MMP-9 and ColIV in dysplastic oral mucosa group

In dysplastic oral mucosa group, the expression of MMP-2 in the basal cell layer was increased compared to normal tissue (MMP-2: iOD 134.69 ± 37.08, Figure 1D). The basal cell layer showed significantly increased MMP-9 immunoreactivity, which was stronger than MMP-2 expression (MMP-9: iOD 307.13 ± 93.22, Figure 1E). The expression of ColIV in the BM was not continuous linear or fragmented (ColIV: iOD 247.83 ± 42.30, Figure 1F, Additional file 1: Figure S1 B).

### The expression of MMP-2, MMP-9 and ColIV in OTSCC tissue group

In the OTSCC tissues, MMP-2 expression was mainly observed in the stromal cells surrounding the epithelial nests of carcinoma (MMP-2: iOD 357.79 ± 116.78; Figure 1G). In some well-differentiated nests of carcinomas, we found keratinization was distinct and the cancer cells were arranged sparsely. The expression of MMP-2 was also negative or weak positive (Figure 1J).

The characteristic distribution pattern of MMP-9 showed a diffuse expression in tumour and stromal cells (MMP-9: iOD 791.31 ± 260.52; Figure 1H). Moreover, MMP-9 positive cells were accumulated around the blood vessels (Figure 1K). Thus, ColIV deposited surrounding cancer nests and formed membrane-like structures in tumour tissue. However, membrane-like structure fragmented, collapsed or even completely disappeared in most cases (ColIV: iOD 151.92 ± 38.17, Figure 1I, Additional file 1: Figure S1 C). Complete membrane-like structure could be observed only in small cases, but it became thick and sparse (Figure 1L).

### Association between MMP-2, MMP-9 and ColIV expression and clinic-pathological characteristics of tongue cancer

As shown in Table 2, tumour MMP-2 expression was only detected in 14 of 48 specimens (low expression in 57% and high expression in 43%). However, for stromal MMP-2 expression, low positivity was noted in 40% of cases, whereas 60% showed high positivity. The presence of tumour MMP-2 expression was associated with differentiation and clinical stage. However, high stromal MMP-2 expression was only associated with positive lymph node status (P < 0.01).

**Table 2:** Relationship between MMP-2, MMP-9 and type IV collagen expression and clinic-pathological parameters in 48 patients with tongue carcinoma

**Variable**		**MMP-2**						**MMP-9**						**Type IV collagen**		
		**Stromal cells**		**P**	**Tumour cells**		**P**	**Stromal cells**		**P**	**Tumour cells**		**P**	**Low**	**High**	**P**
		**Low**	**High**		**Low**	**High**		**Low**	**High**		**Low**	**High**				
Gender	Male	14	22	1.000	31	5	1.000	6	30	0.672	11	25	1.000	24	12	0.139
	Female	5	7		11	1		3	9		4	8		11	1	
Age	<55	9	12	0.683	18	3	1.000	5	16	0.477	5	16	0.327	17	4	0.269
	≥55	10	17		24	3		4	23		10	17		18	9	
Differentiation	Advanced	11	13	0.2	24	0	0.022^▲^	7	17	0.137	8	16	0.756	15	9	0.104
	Medium/poor	8	16		18	6		2	22		7	17		20	4	
Clinical stage	I+II	12	15	0.435	21	6	0.029^▲^	8	19	0.058	9	18	0.724	18	9	0.269
	III+IV	7	14		21	0		1	20		6	15		17	4	
T stage	T1+T2	19	26	0.267	40	5	0.336	9	36	1.000	15	30	0.542	32	13	0.553
	T3+T4	0	3		2	1		0	3		0	3		3	0	
Recurrence	No	15	18	0.217	28	5	0.650	6	27	1.000	12	21	0.328	22	11	0.182
	Yes	4	11		14	1		3	12		3	12		13	2	
Lymph node involvement	No	10	1	<0.001^★^	11	0	0.313	6	5	0.002^★^	8	3	0.002^★^	5	6	0.048^▲^
	Yes	9	28		31	6		3	34		7	30		30	7	

^▲^Correlation was significant at the 0.05 level (two-tailed).

^★^Correlation was significant at the 0.01 level (two-tailed).

Some level of MMP-9 expression was detected in the cytoplasm of the majority of the samples; 69% (33 of 48) of the cases showed high tumour MMP-9 expression (moderate or strong), while only 4 of 48 cases (8%) tested negative for MMP-9 expression. In all the specimens, stromal MMP-9 expression was detected, with 81% showing high expression. High expression of tumour and stromal MMP-9 were significantly associated with positive lymph node status (P < 0.01).

High ColIV expression was observed in 73% (35 of 48) of the samples. Col IV expression was associated with positive lymph node status (P < 0.05), and Spearman’s analysis revealed that the expressions of MMP-2 and MMP-9 were negatively correlated with ColIV expression (P < 0.01 and P < 0.001,respectively; Table 3).

**Table 3:** Association between expressions of MMP-2/MMP-9 and type IV collagen in patients with oral tongue cancer using Spearman’s correlation analysis

**Molecule**		**Type IV collagen**
MMP-2	R	−0.365*
MMP-9	R	−0.568*

R represents the coefficient of correlation.

* Correlation was significant at the 0.05 level (two-tailed).

### Correlation of MMP-2, MMP-9 and ColIV expression with patient survival by univariate analysis

**Figure 2:**
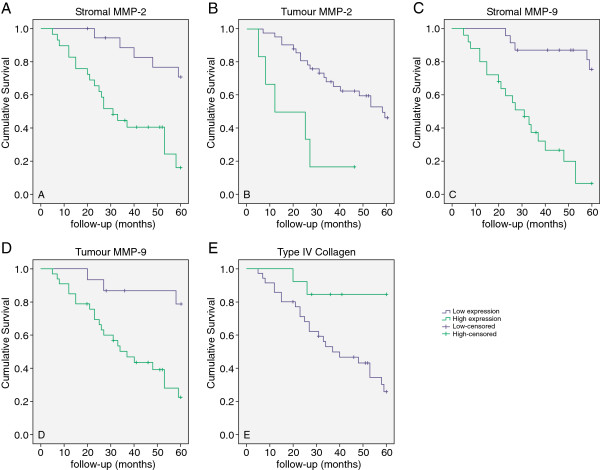
**Kaplan-Meier survival curves for stromal and tumour expression of MMP-2 (A and B), MMP-9 (C and D) and ColIV (E).** The high expression of MMP-2, MMP-9, and type IV collagen (low and high) in tumour was significantly associated with shorter OS (P < 0.001). All samples were positive for stromal MMP-9. Patients with moderate or less expression of stromal MMP-9 have longer OS compared with those with strong expression.

Univariate analysis showed a statistically significant negative correlation between MMP-2 expression in the tumour cells and overall survival (Figure 2A–B), i.e. patients with high MMP-2 expression had a shorter survival than patients with low MMP-2 expression. The same result was observed for a subgroup of patients with MMP-9 positive (P < 0.001) (Figure 2C–D). In contrast, the relationship between overall survival and ColIV expression was inverse (P < 0.01) (Figure 2E), i.e. patients with low ColIV expression had a shorter survival than did patients with high ColIV expression.

## Discussion

The distribution of ColIV in the BM of normal tongue mucosa is compatible with its corresponding functions. When pathological stimulating factors act on tongue mucosa, ColIV attached to the BM can effectively prevent harmful substances from penetrating the BM to the lamina propria [19-21]. Our present study shows, ColIV gradually reduced, was fragmented, collapsed, or even dissolved completely, thus providing channels for cancer cells to invade the lamina propria. ColIV also formed membrane-like structures in tumour tissue, but it became thick and sparse. In well-differentiated carcinomas, we observed that the thick and sparse ColIV around the cancer nests. In moderately and poorly differentiated tumours, the ColIV was destructed around cancer nests. The BM around the cancer nests can restrict tumour invasion and metastasis [10]. So we believe that the well-differentiated tumours may have low malignant potential and weak invasiveness, while, the moderately and poorly differentiated carcinomas have high malignant potential and strong invasiveness. As a result, the massive dissolution of collagen fibers accelerates malignant progression of tumours. In this study, statistical analyses of ColIV showed that changes in their morphological were correlated with progression and differentiation of OTSCC, and with the prognosis of the patients. These results were consistent with Krecicki's findings [19].

It is recognized that carcinomatous invasion is regulated not only by intrinsic genetic changes in cancer cells as the ‘initiators’ of carcinogenesis but also by stromal cell that act as ‘promoters’ [22,23]. Interaction or synergy between tumour cells and stromal cells in the surrounding microenvironment (particularly, between tumour cells and stromal fibroblasts [24-26] and/or monocytes/macrophages [27,28]) can promote tumour spread. This study showed that high MMP expression was found not only in tumour cells but also in stromal cells such as macrophages and vascular endothelial cells. As tumours progress, stromal cells secrete MMPs that can degrade BM and ECM; they can also facilitate tumour spread via interaction with tumour cells. Therefore, stromal cells’ role in tumour progression is of equal importance to that of tumour cells. We also found that patients with high MMP expression in the stromal cells tended to have poorer survival, as high MMP expression is closely tied to lymphatic metastasis. These findings are consistent with the previous studies [29-32]. High MMP-2 or MMP-9 expression in tumour or stromal cells might serve as prognostic predictors. Research on interaction between tumour cells and stromal cells aids further understanding of OTSCC invasiveness from aspects besides genetic mutation.

Our study also showed that expression of MMP-2 and MMP-9 are differentiated among tumours. As tumours progressed, MMP-9 expression increased in tumour epithelium and stroma, while the changes in MMP-2 expression in tumour cells was not as obvious as MMP-9. Double staining of the OTSCC indicated a co-localization of MMP-9 and PCNA (see Additional file 1: Figure S2); correlation analysis showed MMP-9 expression to be positively correlated with that of PCNA (see Additional file 2: Table S1). In other words, expression of MMP-9 protein was significantly increased in tongue cancer cells with strong proliferative ability, although such correlation was not significant for MMP-2. In blood vessels with high MMP-9 expression, ColIV in vascular basement membranes showed certain defects, or the BM became thin. Blood vessels without MMP-9 accumulation had no obvious changes in BM structure. Therefore, the MMP-9 expression may be closely related to proliferation, invasion and metastasis of tumour cells, and even to tumour angiogenesis. Yasumitsu et al. [33] determined gelatinase activity in human schwannoma YST-3 cell lines using zymography, and found that MMP-9 activity in degrading collagen was about 25 times that of MMP−2. Previous studies suggested that MMP-9 expression were closely related to tumour angiogenesis than MMP-2 [34,35].

## Conclusion

Obviously, tumour cells and stromal cells can expression high MMP levels, which are closely related to poor prognosis. In exploring ColIV expression, we also found that tumour expressions of MMP-2 and MMP-9 showed certain variations. The MMP-9 expression may be closely related to proliferation, invasion, and metastasis of tumour cells, and even to tumour angiogenesis. This may be related to the activity of MMP-9; however, its specific mechanism of action merits further research. In addition, which specific stromal cell (e.g. macrophages, fibroblasts, etc.) and which cell subtype (e.g. M1 and M2 macrophages) interact with tumour cells also remains unknown. Nevertheless, clinical application of agents that may inhibit MMP-9 secretion by stromal cells may be a key to achieving clinical control of invasion and metastasis of oral tumours.

## Competing interests

The authors declare that they have no competing interests.

## Authors' contributions

HXF and HXL conceived and designed the experiments. HXF and HXL performed the experiments and analyzed the data. ZXZG contributed to the acquisition of the data, DC has made substantial contribution to collected tissue samples, and HXF, HXL, and JHZ wrote the manuscript. All authors have read and approved the final manuscript.

## Supplementary Material

Additional file 1 **Immunofluorescence staining for ColIV, MMP-9 and PCNA in OTSCC.Figure S1** Immunofluorescence staining for ColIV in normal group, dysplastic oral mucosa group and OTSCC group. Comparative immunolocalization of ColIV in normal group, dysplastic oral mucosa group and OTSCC (T and S indicate the tumour and stroma respectively) by immunofluorescence. **(A)** The expression of ColIV in the BM of normal group showing linear and continuous marking (red arrow). **(B)** The expression of ColIV in the BM of normal group showing interrupted (red arrow). **(C)** In the OTSCC, the expression of ColIV are showed fragmented or collapsed (red arrow). Original magnification, 200×. **Figure S2** Double immunofluorescence staining for PCNA and MMP-9 in the stromal of OTSCC. Expression of PCNA and MMP-9 proteins detected by double immunofluorescence staining in the stromal of OTSCC (S indicate the stroma). **(A)** The expression of PCNA in the stromal cells (red). **(B)** The expression of MMP-9 in the stromal cells (green). **(C)** Double-labeled cells of PCNA/MMP-9 in the OTSCC. Original magnification, 200×. Click here for file

Additional file 2 **Table S1.** Association between MMP-2 and MMP-9 expression and PCNA in OTSCC patients. Click here for file
