# Supplementary material for: Changes in the expression of MMP2, MMP9, and ColIV in stromal cells in oral squamous tongue cell carcinoma: relationships and prognostic implications
Source: J Exp Clin Cancer Res. 2012 Oct 29;31(1):90. doi: 10.1186/1756-9966-31-90 (PMC3490717; doi:10.1186/1756-9966-31-90)
Supplement: Additional file 1 — Immunofluorescence staining for ColIV, MMP-9 and PCNA in OTSCC.Figure S1 Immunofluorescence staining for ColIV in normal group, dysplastic oral mucosa group and OTSCC group. Comparative immunolocalization of ColIV in normal group, dysplastic oral mucosa group and OTSCC (T and S indicate the tumour and stroma respectively) by immunofluorescence. (A) The expression of ColIV in the BM of normal group showing linear and continuous marking (red arrow). (B) The expression of ColIV in the BM of normal group showing interrupted (red arrow). (C) In the OTSCC, the expression of ColIV are showed fragmented or collapsed (red arrow). Original magnification, 200×. Figure S2 Double immunofluorescence staining for PCNA and MMP-9 in the stromal of OTSCC. Expression of PCNA and MMP-9 proteins detected by double immunofluorescence staining in the stromal of OTSCC (S indicate the stroma). (A) The expression of PCNA in the stromal cells (red). (B) The expression of MMP-9 in the stromal cells (green). (C) Double-labeled cells of PCNA/MMP-9 in the OTSCC. Original magnification, 200×. [file 1756-9966-31-90-S1.ppt]

## Slide 1
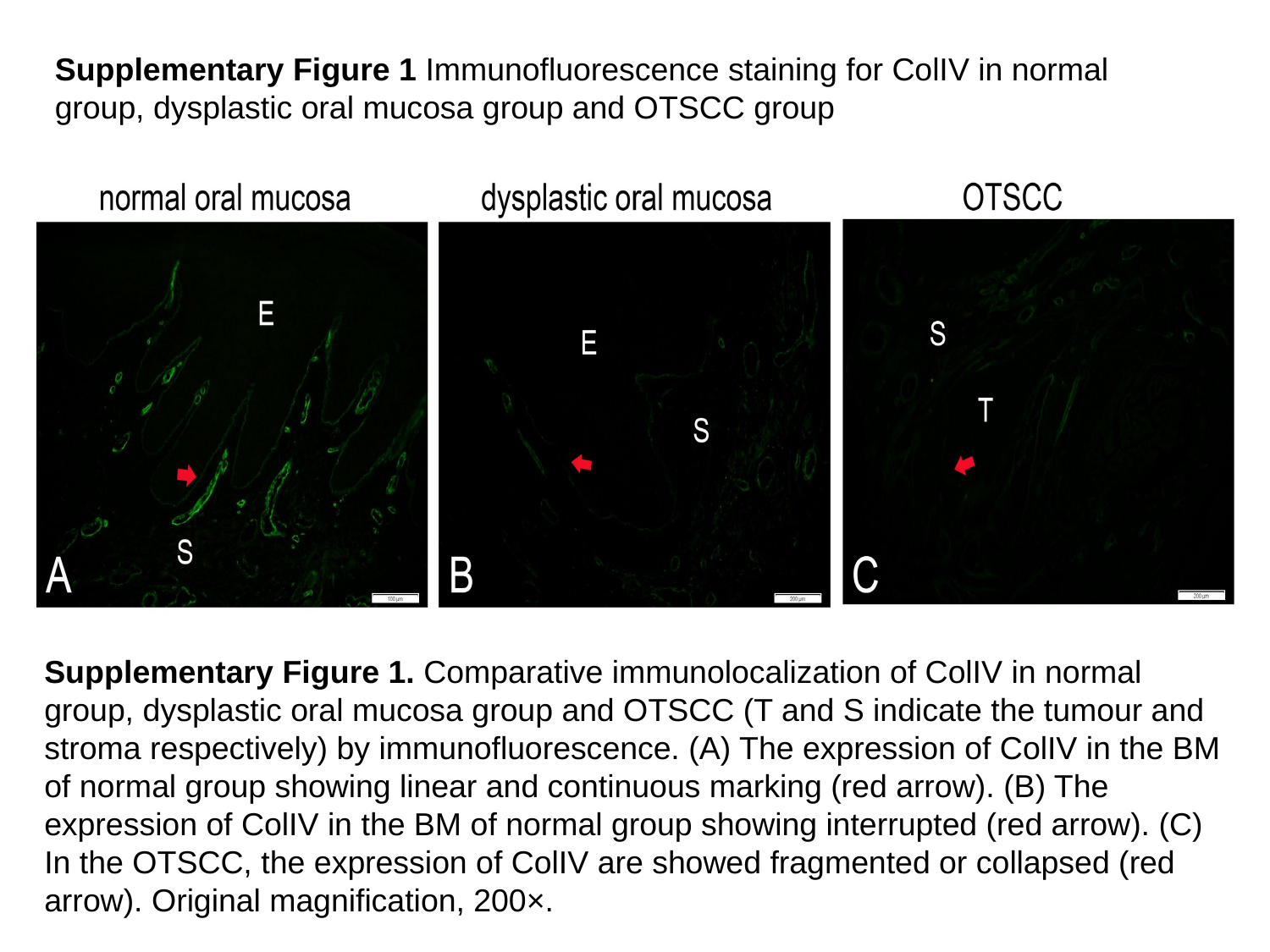

Supplementary Figure 1 Immunofluorescence staining for ColIV in normal group, dysplastic oral mucosa group and OTSCC group
Supplementary Figure 1. Comparative immunolocalization of ColIV in normal group, dysplastic oral mucosa group and OTSCC (T and S indicate the tumour and stroma respectively) by immunofluorescence. (A) The expression of ColIV in the BM of normal group showing linear and continuous marking (red arrow). (B) The expression of ColIV in the BM of normal group showing interrupted (red arrow). (C) In the OTSCC, the expression of ColIV are showed fragmented or collapsed (red arrow). Original magnification, 200×.

## Slide 2
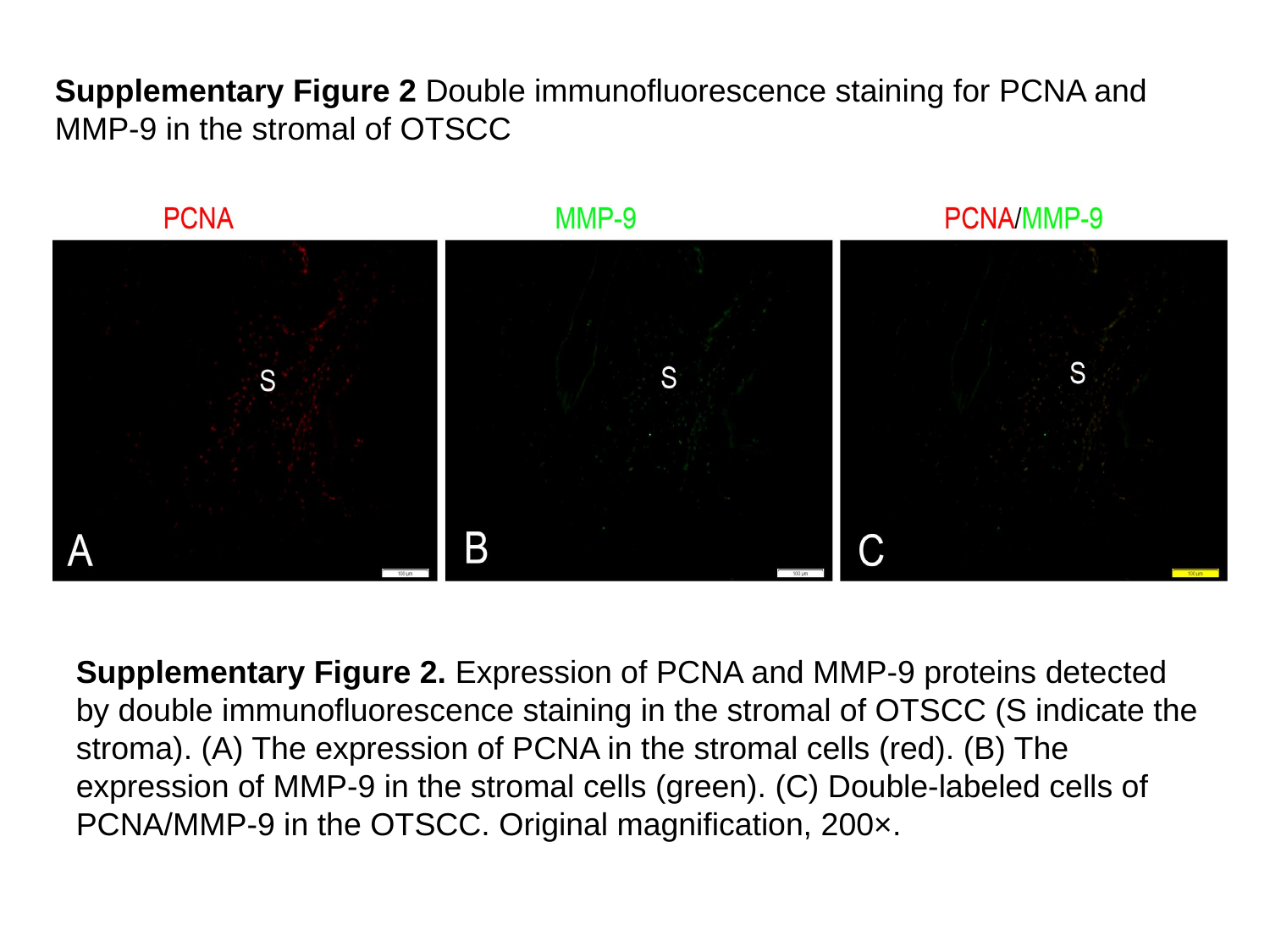

Supplementary Figure 2 Double immunofluorescence staining for PCNA and MMP-9 in the stromal of OTSCC
Supplementary Figure 2. Expression of PCNA and MMP-9 proteins detected by double immunofluorescence staining in the stromal of OTSCC (S indicate the stroma). (A) The expression of PCNA in the stromal cells (red). (B) The expression of MMP-9 in the stromal cells (green). (C) Double-labeled cells of PCNA/MMP-9 in the OTSCC. Original magnification, 200×.
