# Supplementary material for: Changes in the expression of MMP2, MMP9, and ColIV in stromal cells in oral squamous tongue cell carcinoma: relationships and prognostic implications
Source: J Exp Clin Cancer Res. 2012 Oct 29;31(1):90. doi: 10.1186/1756-9966-31-90 (PMC3490717; doi:10.1186/1756-9966-31-90)
Supplement: Additional file 2 — Table S1. Association between MMP-2 and MMP-9 expression and PCNA in OTSCC patients. [file 1756-9966-31-90-S2.doc]

**Supplementary Table 1. Association between MMP-2 and MMP-9 expression and PCNA in OTSCC patients**

| Molecule |  | PCNA |
| --- | --- | --- |
| MMP-2 | R | 0.263 |
| MMP-9 | R | 0.515* |

Data was analyzed using Spearman’s correlation analysis.

R represents the coefficient of correlation.

*Correlation was significant at the 0.01 level (two-tailed).
